# Non-contiguous finished genome sequence and description of *Kurthia senegalensis* sp. nov.

**DOI:** 10.4056/sigs.5078947

**Published:** 2014-02-20

**Authors:** Véronique Roux, Jean-Christophe Lagier, Aurore Gorlas, Catherine Robert, Didier Raoult

**Affiliations:** 1 Aix Marseille Université, URMITE, Faculté de médecine, Aix-Marseille Université, Marseille, France

**Keywords:** Kurthia senegalensis, *Firmicutes*, capsule, flagella, culturomics

## Abstract

*Kurthia senegalensis* strain JC8E^T^ sp. nov. is the type strain of *K. senegalensis* sp. nov., a new species within the genus Kurthia. This strain, whose genome is described here, was isolated from the fecal flora of a healthy patient. *K. senegalensis* is an aerobic rod. Here we describe the features of this organism, together with the complete genome sequence and annotation. The 2,975,103 bp long genome contains 2,889 protein-coding genes and 83 RNA genes, including between 4 and 6 rRNA genes.

## Introduction

*Kurthia senegalensis* strain JC8E^T^ (CSUR P138^T^ = DSM 24641^T^) is the type strain of *K. senegalensis* sp. nov. This bacterium is a Gram-positive strictly aerobic rod, capsulated, motile by peritrichous flagella and was isolated from the stool of a healthy Senegalese patient as part of a "culturomics" study aiming at cultivating individually all species within human feces [1].

Presently, "the gold standard method" to define a bacterial species is DNA-DNA hybridization (DDH) [2]. But this method is time consuming and the inter-laboratory reproducibility is poor. So, with the development of PCR and sequencing methods, 16S rRNA gene sequence comparison is often the preferred approach for recognizing a new taxon when a gene sequence similarity less than 97% is found [3]. To make descriptions more complete, phenotypic criteria (morphology, biochemical tests, growth conditions, chemotaxonomy) have to be included to characterize a prokaryote strain [4]. Fortunately, sequencing whole prokaryote genomes is now possible for more laboratories, and descriptions of sequencing protocols should be included in all species descriptions. Such activity would supplant the need for most other methods used during genome annotation, and new bioinformatics methods based on genome-to-genome comparison have been proposed to replace the DDH approach [5].

Here we present a summary classification and a set of features for *K. senegalensis* sp. nov. strain JC8E^T^ together with the description of the complete genomic sequencing and annotation. These characteristics support the circumscription of the species *K. senegalensis*.

Kurth described *Bacterium zopfii*, isolated from the intestinal contents of chickens, which became later the first species of the genus Kurthia*,* K. zopfii. The genus Kurthia was created in 1885 by Trevisan [6] in honor of Kurth. The name Kurthia was first published in the seventh edition of *Bergey’s Manual of Determinative Bacteriology* [7]. Currently, the genus includes 4 species: K. zopfii, K. gibsonii [8], K. sibirica [9] and K. massiliensis [10]. The bacteria are included in the Firmicutes phylum, in the Planococcaceae family.

## Classification and features

**Figure 1:**
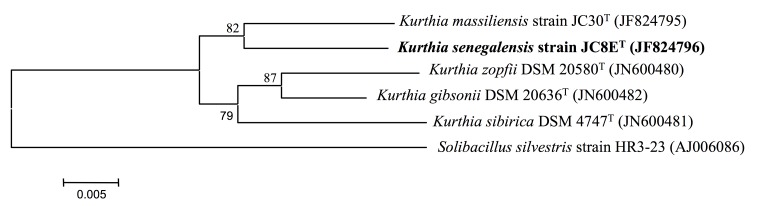
Phylogenetic tree highlighting the position of *Kurthia senegalensis* strain JC8E^T^ relative to other type strains within the Kurthia genus. GenBank accession numbers are indicated in parentheses. Sequences were aligned using CLUSTALX, and phylogenetic inferences obtained using the neighbor joining method as implemented in the MEGA 5 software package [24]. Numbers at the nodes are percentages of bootstrap values supporting that node using 1,000 bootstrap replicates to generate a majority consensus tree. Solibacillus silvestris was used as the outgroup. The scale bar represents 0.005 nucleotide change per nucleotide position.

A stool sample was collected from a healthy 16-year-old male Senegalese volunteer patient living in Dielmo (a rural village in the Guinean-Sudanian zone in Senegal), who was included in a research protocol. The patient gave an informed and signed consent, and the agreement of the National Ethics Committee of Senegal and the local ethics committee of the IFR48 (Marseille, France) were obtained under agreement 09-022). The fecal specimen was preserved at -80°C after collection and sent to Marseille. Strain JC8E (Table 1) was isolated in January 2011 by aerobic cultivation on 5% sheep blood-enriched Columbia agar (BioMerieux). There is no evidence of pathogenicity for the strain. JC8E exhibited a 96.8% nucleotide sequence similarity with K. massiliensis, the phylogenetically closest validated Kurthia species (Figure 1). This value was lower than the 97% 16S rRNA gene sequence threshold to delineate a new species without carrying out DNA-DNA hybridization recommended by the report of the *ad hoc* committee on reconciliation of approaches to bacterial systematics [2]. Moreover, Stackebrandt and Ebers proposed to increase this value to 98.7% [25]. Recently, Auch *et al*. proposed a genome-to-genome comparison approach to replace the DDH approach [5]. As we sequenced the genomes of K. massiliensis and *K. senegalensis,* we tested this new approach. We chose GGDC 2.0 Blast + as the alignment method for finding intergenomic matches, using a formula based on the number of identities divided by the HSP (High scoring Segment Pairs) length to infer distances. The DDH estimate resulted from a generalized linear model (GLM). The GLM-based DDH estimate was 21% ± 2.33. The found value (< 70%) confirmed that K. massiliensis and isolate JC8E^T^ did not belong to the same species.

**Table 1:** Classification and general features of *Kurthia senegalensis* strain JC8E^T^ [11]

**MIGS ID**	**Property**	**Term**	**Evidence code^a^**
	Current classification	Domain Bacteria	TAS [12]
		Phylum Firmicutes	TAS [13-15]
		Class Bacilli	TAS [16,17]
		Order Bacillales	TAS [18,19]
		Family Planococcaceae	TAS [19,20]
		Genus Kurthia	TAS [6,19,21,22]
		Species *Kurthia senegalensis*	IDA
		Type strain JC8E^T^	IDA
	Gram stain	Positive	IDA
	Cell shape	Coccobacilli	IDA
	Motility	Motile by peritrichous flagella	IDA
	Sporulation	Nonsporulating	IDA
	Temperature range	Mesophile	IDA
	Optimum temperature	37°C	IDA
MIGS-6.3	Salinity	Growth in BHI medium + 2% NaCl	IDA
MIGS-22	Oxygen requirement	Aerobic	IDA
	Carbon source	Unknown	NAS
	Energy source	Unknown	NAS
MIGS-6	Habitat	Human gut	IDA
MIGS-15	Biotic relationship	Free living	IDA
MIGS-14	Pathogenicity	Unknown	NAS
	Biosafety level	2	
	Isolation	Human feces	
MIGS-4	Geographic location	Senegal	IDA
MIGS-5	Sample collection time	September 2010	IDA
MIGS-4.1	Latitude	13.7167	IDA
MIGS-4.1	Longitude	16.4167	IDA
MIGS-4.3	Depth	Surface	IDA
MIGS-4.4	Altitude	51 m above sea level	IDA

Evidence codes - IDA: Inferred from Direct Assay; TAS: Traceable Author Statement (i.e., a direct report exists in the literature); NAS: Non-traceable Author Statement (i.e., not directly observed for the living, isolated sample, but based on a generally accepted property for the species, or anecdotal evidence). These evidence codes are from the Gene Ontology project [23]. If the evidence is IDA, then the property was directly observed for a live isolate by one of the authors or an expert mentioned in the acknowledgements.

**Figure 2:**
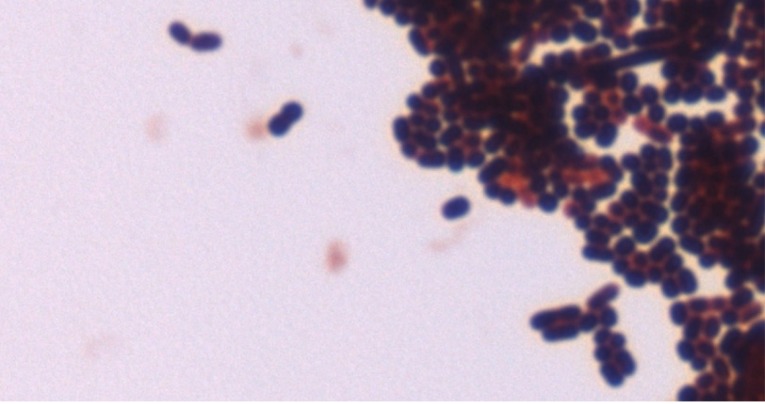
Gram stain of *K. senegalensis* strain JC8E^T^

Surface colonies were observed on sheep blood agar (bioMérieux) after 24 hours aerobic incubation at 37°C. The colonies of the strain JC8E^T^ were yellowish, mat, flat and spread, 5 mm in diameter. Gram staining showed Gram-positive coccobacilli (Figure 2).

Six different growth temperatures (25, 30, 37, 45, 50 and 55°C) were tested. Growth occurred between 25°C and 50°C, and optimal growth was observed between 30°C and 50°C. Growth of the strain was tested under an aerobic atmosphere, in the presence of 5% CO_2_, and also in anaerobic and microaerophilic atmospheres which were created using GENbag anaer and GENbag microaer (bioMérieux), respectively. The strains were aerobic and also grew under microaerophilic conditions and in the presence of 5% CO_2_ but did not grow in an anaerobic atmosphere. The NaCl concentrations allowing growth of strain JC8E^T^, were determined on Difco^TM^Brain Heart Infusion Agar plates (Becton Dickinson). The powder was supplemented with NaCl (Euromedex) to obtain the tested concentrations (0.5, 1, 2, 3, 5 10, 15%, w/v). Growth occurred between 0.5-5% NaCl but the optimum growth was between 0.5-2% NaCl.

Growth in the range of pH 5.0-10.0 was tested using BBL^TM^ Brain Heart Infusion (Becton Dickinson). Final pH was adjusted with HCl or NaOH solution. Growth occurred between pH 5-9.

**Figure 3:**
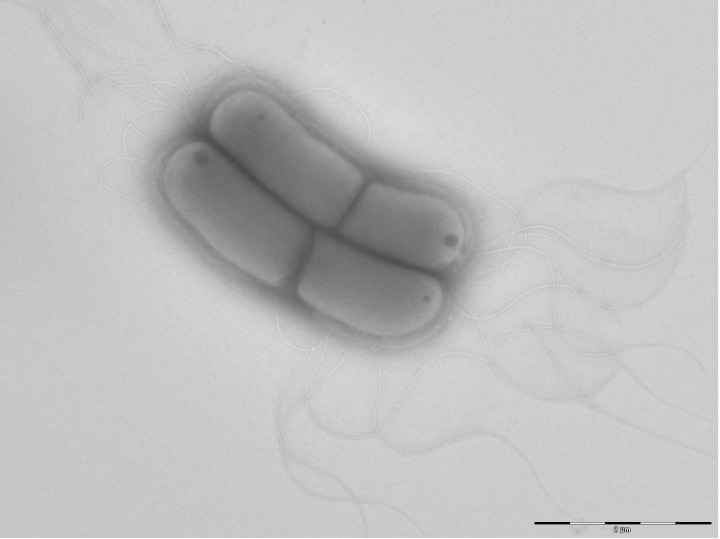
Transmission electron micrograph of *K. senegalensis* strain JC8E^T^, using a Morgani 268D (Philips) at an operating voltage of 60kV.The scale bar represents 2 μm.

**Figure 4:**
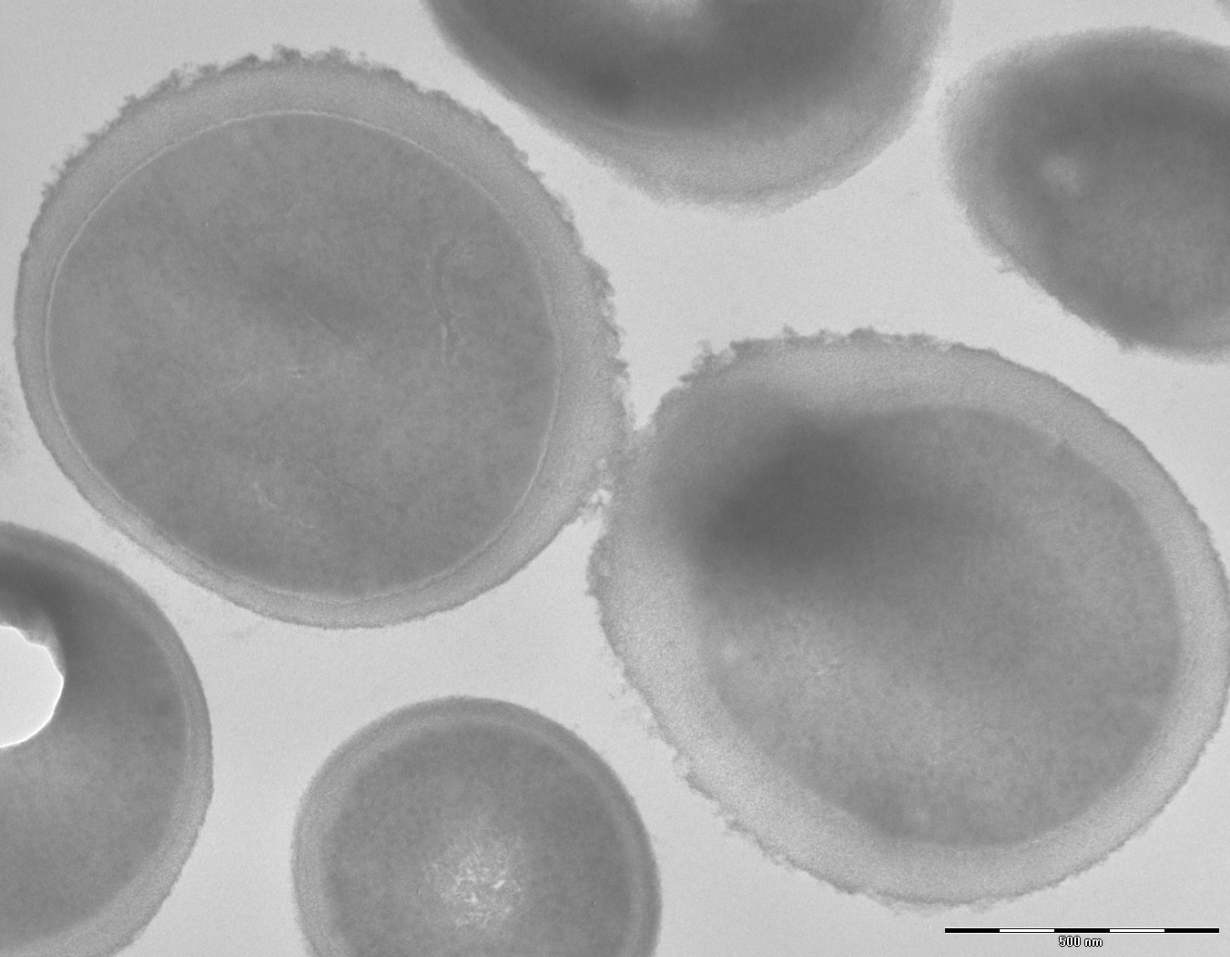
Capsule characterization of *K. senegalensis* after the bacteria were embedded in Epon 812 resin and observed by transmission electron microscopy.

The size and ultrastructure of cells were determined by negative staining transmission electron microscopy. The rods were 1.8-9.2 μm long and 0.7-1.2 μm wide (Figure 3). Peritrichous flagella were observed. Capsule was characterized by India ink stain and after the bacteria were embedded in Epon 812 resin and observed by transmission electron microscopy (Figure 4).

Strain JC8E^T^ exhibited catalase activity but no oxidase activity. Api ZYM, Api 20NE (BioMérieux) were used to study biochemical characters (Table 2).

**Table 2:** Diagnostic traits differentiating five Kurthia species.

**Characteristic**	1	**2**	**3**	**4**	**5**
gelatin hydrolysis	+	w	-	-	-
N-acetyl-glucosamine assimilation	-	+	-	+	-
D-maltose assimilation	+	-	-	-	-
potassium gluconate assimilation	+	+	-	-	-
capric acid assimilation	-	+	-	-	-
trisodium citrate assimilation	+	-	-	-	-
alkaline phosphatase	-	+	+	w	+
esterase (C4)	+	+	+	w	w
esterase lipase (C8)	+	+	+	w	w
valine arylaminidase	w	-	-	+	-
cystine arylaminidase	+	-	+	-	-
trypsin	-	w	-	-	-
α-chemotrypsin	w	w	-	+	-
naphthol-AS-BI-phosphohydrolase	-	-	-	-	+
α-glucosidase	+	-	-	-	-

Strains:1, K massiliensis JC30^T^ ; 2, *K. senegalensis* sp. nov. JC8E^T^ 3, K. gibsonii DSM 20636^T^; 4, K. zopfii DSM 20580^T^; 5, K. sibirica DSM 4747^T^.

+: positive result, -: negative result, w: weak positive result

Analysis of respiratory quinones by HPLC was carried out by the Identification Service and Dr Brian Tindall, DSMZ, Braunschweig, Germany. Respiratory lipoquinones were extracted from 100 mg of freeze dried cell material as described by Tindall [26,27]. Respiratory lipoquinones were separated into their different classes (menaquinones and ubiquinones) by thin layer chromatography on silica gel, using hexane:*tert*-butylmethylether (9:1 v/v) as solvent. UV absorbing bands corresponding to menaquinones or ubiquinones were removed from the plate and further analyzed by HPLC at 269 nm. The respiratory quinones were MK-7 (100%) for strain JC8E^T^. Preparation and determination of cellular fatty acids were carried out by following the procedures given for the Sherlock Microbial identification System (MIDI). The major fatty acids were C15:0 iso 50.75% and C15:0 anteiso 24.05%. Polar lipids were extracted from 100 mg of freeze dried cell material using a chloroform:methanol:0.3% aqueous NaCl mixture 1:2:0.8 (v/v/v) (modified after [28]). The extraction solvent was stirred overnight and the cell debris pelleted by centrifugation. Polar lipids were recovered into the chloroform phase by adjusting the chloroform:methanol:0.3% aqueous NaCl mixture to a ratio of 1:1:0.9 (v/v/v). Polar lipids were separated as previously described [29]. The polar lipids present were diphosphatidylglycerol, phosphatidylglycerol, phosphatidylethanolamine, phospholipids 1 and 2, unidentified aminophospholipid and glycolipid. The peptidoglycan of the bacteria was isolated as described by Schleifer [30]. The determination was carried out as previously described [30,31] with the modification that TLC on cellulose was applied instead of paper chromatography. Quantitative analysis of amino acids was performed after derivatization by gas chromatography and gas chromatography / mass spectrometry (320-MS Quadrupole GC/MS, Varian) [32]. *K. senegalensis* showed the peptidoglycan type A4αL-Lys←D-Glu (type A11.33 according to ref [33]).

*K. senegalensis* was susceptible to penicillin G, amoxicillin, amoxicillin plus clavulanic acid, imipenem, gentamycin, erythromycin, doxycycline, rifampicin, vancomycin, nitrofurantoin. It was resistant to ceftriaxone, ciprofloxacin, sulfamethoxazole trimethoprim and metronidazole.

**Figure 5:**
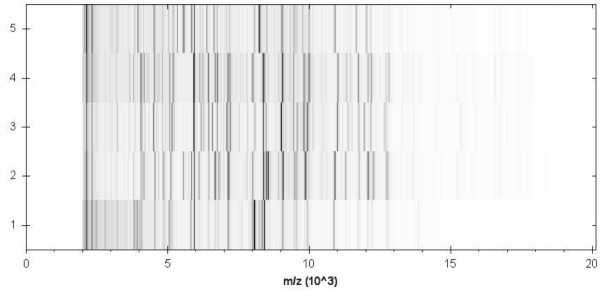
Reference mass spectra from *K. senegalensis* strain JC8E^T^ and other representatives of the genus Kurthia*.* Spectra from 24 individual colonies were compared and a reference spectrum was generated. Strains: 1, K massiliensis JC30^T^ ; 2, *K. senegalensis* sp. nov. JC8E^T^; 3, K. sibirica DSM 4747^T^; 4, K. gibsonii DSM 20636^T^; 5, K. zopfii DSM 20580^T^.

Matrix-assisted laser-desorption/ionization time-of-flight (MALDI-TOF) MS protein analysis was carried out. Briefly, a pipette tip was used to pick one isolated bacterial colony from a culture agar plate, and to spread it as a thin film on a MALDI-TOF target plate (Bruker Daltonics). Twelve distinct deposits were done for strain JC8E^T^ from twelve isolated colonies and the manipulation was repeated another day. After air-drying, 1.5 µl matrix solution (saturated solution of α-cyanohydroxycinnaminic acid in 50% aqueous acetonitrile containing 2.5% trifluoroacetic acid) per spot was applied. MALDI-TOF MS was conducted using the Microflex LT spectrometer (Bruker Daltonics). All spectra were recorded in linear, positive ion mode. The acceleration voltage was 20 kV. Spectra were collected as a sum of 240 shots across a spot. Preprocessing and identification steps were performed using the manufacturer’s parameters. The JC8E^T^ spectra were imported into the MALDI BioTyper software (version 3.0, Bruker) and analysed by standard pattern matching (with default parameter settings) against the main spectra of 6,300 bacteria, including the spectra from K. gibsonii, K. sibirica*,* K. zopfii and K. massiliensis, in the BioTyper database. A score enabled the identification, or not, from the tested species: a score > 2.3 with a validated species enabled the identification at the species level, a score > 1.7 but < 2 enabled the identification at the genus level; and a score < 1.7 did not enable any identification. For strain JC8E^T^, none of the obtained scores were > 1, thus suggesting that our isolate was not a member of a known species. We added the spectrum from strain JC8E^T^ to our database (Figure 5). The spectrum is available online in our free-access URMS database.

## Genome sequencing information

### Genome project history

The organism was selected for sequencing on the basis of its phylogenetic position and 16S rDNA similarity to other members of the genus Kurthia, and is part of a “culturomics” study of the human digestive flora aiming at isolating all bacterial species within human feces. It was the second genome of a Kurthia species, *Kurthia senegalensis* sp. nov. A summary of the project information is shown in Table 3. The EMBL accession number is CAEW01000000 and consists of 46 contigs (≥500 bp) and 17 scaffolds (> 2,575 bp). Table 3 shows the project information and its association with MIGS version 2.0 compliance.

**Table 3:** Project information [11]

**MIGS ID**	**Property**	**Term**
MIGS-31	Finishing quality	High-quality draft
MIGS-28	Libraries used	One paired end 3-kb library and one Shotgun library
MIGS-29	Sequencing platforms	454 GS FLX Titanium
MIGS-31.2	Fold coverage	23×
MIGS-30	Assemblers	Newbler version 2.5.3
MIGS-32	Gene calling method	Prodigal
	EMBL ID	CAEW01000000
	EMBL Date of Release	February 28, 2012
	Project relevance	Study of the human gut microbiome

### Growth conditions and DNA isolation

*K. senegalensis* sp. nov. strain JC8E^T^, CSUR P138^T^, DSM 24641^T^, was grown aerobically on 5% sheep blood-enriched Columbia agar at 37°C. 3 petri dishes were spread and resuspended in 3×100µl of G2 buffer. A first mechanical lysis was performed with glass powder on the Fastprep-24 device (Sample Preparation system) from MP Biomedicals, USA using 2×20 second bursts. DNA was then treated with lysozyme (30 minutes at 37°C) and extracted using the BioRobot EZ 1 Advanced XL (Qiagen). The DNA was then concentrated and purified on a Qiamp kit (Qiagen). The yield and the concentration was measured by the Quant-it Picogreen kit (Invitrogen) on the Genios Tecan fluorometer at 86 ng/µl.

### Genome sequencing and assembly

Shotgun and 3-kb paired-end sequencing strategies were performed. The shotgun library was constructed with 500 ng of DNA with the GS Rapid library Prep kit (Roche). For the paired-end sequencing, 5 µg of DNA was mechanically fragmented on a Hydroshear device (Digilab) with an enrichment size at 3-4 kb. The DNA fragmentation was visualized using the 2100 BioAnalyzer (Agilent) on a DNA labchip 7500 with an optimal size of 3.679 kb. The library was constructed according to the 454 GS FLX Titanium paired-end protocol. Circularization and nebulization were performed and generated a pattern with an optimal size of 497 bp. After PCR amplification through 15 cycles followed by double size selection, the single stranded paired-end library was then quantified using the Genios fluorometer (Tecan) at 888 pg/µL. The library concentration equivalence was calculated as 3.28 x 10^9^molecules/µL. The library was stored at -20°C until further use.

The shotgun and paired-end libraries were clonally-amplified with 3 cpb and 1cpb in 3 and 4 emPCR reactions respectively on the GS Titanium SV emPCR Kit (Lib-L) v2 (Roche). The yields of the emPCR were 14.72 and 20% respectively. 340,000 beads for the shotgun application and 790,000 beads for the 3kb paired end were loaded on the GS Titanium PicoTiterPlate PTP Kit 70x75 and sequenced with the GS FLX Titanium Sequencing Kit XLR70 (Roche). The run was performed overnight and then analyzed on the cluster through the gsRunBrowser and Newbler assembler (Roche). A total of 307,968 passed filter wells were obtained and generated 69.7 Mb with a length average of 223 bp. The passed filter sequences were assembled using Newbler with 90% identity and 40 bp as overlap. The final assembly identified 17 scaffolds and 42 large contigs (>1,500 bp).

### Genome annotation

Open Reading Frames (ORFs) were predicted using Prodigal [34] with default parameters but the predicted ORFs were excluded if they were spanning a sequencing GAP region. The predicted bacterial protein sequences were searched against the GenBank database [35] and the Clusters of Orthologous Groups (COG) databases [36] using BLASTP. The tRNAscan-SE tool [37] was used to find tRNA genes, whereas ribosomal RNAs were found by using RNAmmer [38].

Transmembrane domains and signal peptides were predicted using TMHMM [39] and SignalP [40], respectively. ORFans were identified if their BLASTp *E*-value was lower than 1e-03 for alignment length greater than 80 amino acids. If alignment lengths were smaller than 80 amino acids, we used an *E*-value of 1e-05. Such parameter thresholds have been used in previous works to define ORFans.

To estimate the mean level of nucleotide sequence similarity at the genome level between *K. senegalensis* and K. massiliensis (GenBank accession number CAEU01000000), the only available Kurthia genome to date, we compared the ORFs only using comparison sequences found in the server RAST [41] at a query coverage of ≥60% and a minimum nucleotide length of 100 bp.

## Genome properties

**Figure 6:**
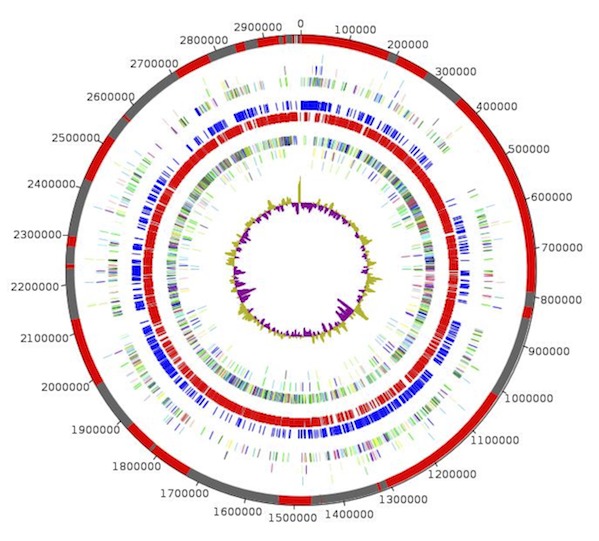
Graphical circular map of *Kurthia senegalensis* genome. From outside to the center: Genes on forward strand (colored by COG categories), genes on reverse strand (colored by COG categories), RNA genes (tRNAs green, rRNAs red), GC content, and GC skew (3 circles).

The genome is 2,975,103 bp long with a 38.21% GC content (Table 3, Figure 6). Of the 2,972 predicted genes, 2,889 were protein-coding genes, and 83 were RNAs. A total of 2,141 genes (74.11%) were assigned a putative function. The remaining genes were annotated as either hypothetical proteins or proteins of unknown function. The distribution of genes into COGs functional categories is presented in Table 4. The properties and the statistics of the genome are summarized in Tables 4 and 5.

**Table 4:** Nucleotide content and gene count levels of the genome

Attribute	Value	% of total^a^
Genome size (bp)	2,975,103	100
DNA G+C content (bp)	1,136,726	38.21
DNA coding region (bp)	2,576,027	86.59
Total genes	2,972	100
RNA genes	83	2.79
Protein-coding genes	2,889	97.21
Genes with function prediction	2,141	74.11
Genes assigned to COGs	2,272	78.64
Genes with peptide signals	335	11.6
Genes with transmembrane helices	67	23.19

a) The total is based on either the size of the genome in base pairs or the total number of protein coding genes in the annotated genome.

**Table 5:** Number of genes associated with the 25 general COG functional categories

**Code**	**Value**	**%age**	**Description**
J	164	5.68	Translation
A	0	0	RNA processing and modification
K	201	6.96	Transcription
L	128	4.43	Replication, recombination and repair
B	1	0.03	Chromatin structure and dynamics
D	34	1.18	Cell cycle control, mitosis and meiosis
Y	0	0	Nuclear structure
V	44	1.52	Defense mechanisms
T	148	5.12	Signal transduction mechanisms
M	116	4.02	Cell wall/membrane biogenesis
N	70	2.42	Cell motility
Z	0	0	Cytoskeleton
W	0	0	Extracellular structures
U	40	1.38	Intracellular trafficking and secretion
O	81	2.80	Posttranslational modification, protein turnover, chaperones
C	117	4.05	Energy production and conversion
G	117	4.05	Carbohydrate transport and metabolism
E	264	9.14	Amino acid transport and metabolism
F	71	2.46	Nucleotide transport and metabolism
H	104	3.60	Coenzyme transport and metabolism
I	109	3.77	Lipid transport and metabolism
P	180	6.23	Inorganic ion transport and metabolism
Q	62	2.15	Secondary metabolites biosynthesis, transport and catabolism
R	378	13.08	General function prediction only
S	222	7.68	Function unknown
X	617	21.36	Not in COGs

The total is based on the total number of protein coding genes in the annotated genome.

## Comparison with other Kurthia genomes

To date, only the genome of K. massiliensis strain JC30^T^ has also been sequenced. *K. senegalensis* strain JC8E^T^ shares a mean sequence similarity of 80.57% (60.06-99.58%) with K. massiliensis JC30^T^. The genome size, the G+C% and the total genes of *K. senegalensis* strain JC8E^T^ are lower than those of K. massiliensis JC30^T^ (Table 6).

**Table 6:** Genome characteristics of Kurthia representatives.

**Attribute**	***K. senegalensis* strain JC8E^T^**	K. massiliensis** JC30^T^**
Genome size (bp)	2,975,103	3,199,090
DNA G+C content (%)	38.21	39.26
Total genes	2,972	3,326
Protein-coding genes	2,889	3,240

## Prophage genome properties

Prophage Finder [42] and PHAST [43] were used to identify potential prophages in *K. senegalensis* strain JC8E^T^ genome. The genome contains at least one genetic element of around 36.3 kb (with a GC content of 38.9%), which we named KS1, on contig 21. The overall G + C content of the KS1 genome (38.9%) is comparable with the overall G + C content of *K. senegalensis* genome (38.21%), allowing KS1 to be maintained and regulated inside the host [44].

A total of 49 open reading frames (ORFs) larger than 98 nucleotides were recovered from KS1, and most of them (24) encode proteins sharing a high identity with proteins found in Bacillales genus phages. The majority of the putative genes (43) have the same orientation and six are located on the complementary strand. Preliminary annotation of KS1 was performed and the majority of the putative genes (31) encode hypothetical proteins. The 19 ORFs with an attributed function encode proteins involved in DNA packaging, head and tail morphogenesis structure, cell lysis and lysogeny control, DNA replication, recombination, and modification.

## Conclusion

On the basis of phenotypic, phylogenetic and genomic analyses, we formally propose the creation of *Kurthia senegalensis* sp. nov. that contains the strain JC8E^T^. This strain originated in Senegal.

### Description of *Kurthia senegalensis* sp. nov.

*Kurthia senegalensis* (se.ne.gal.e’n.sis, L. gen. masc. n. senegalensis pertaining to Senegal, the country where the type strain was isolated). Isolated from stool of a healthy Senegalese patient. *K senegalensis* are aerobic Gram-positive coccobacilli. Surface colonies were observed on sheep blood agar after 24 h aerobic incubation at 37°C. The colonies of the strain JC8E^T^ were circular, greyish/yellowish, shiny, curved and smooth, 2-5 mm in diameter. Cells are motile by peritrichous flagella and capsulated. Catalase activity is positive but oxidase activity is negative. Gelatine hydrolysis, N-acetyl-glucosamine assimilation, potassium gluconate assimilation, capric acid assimilation and malic acid assimilation are present. Alkaline phosphatase, esterase (C4), esterase lipase (C8), leucine arylaminidase, trypsin, α-chemotrypsin and acid phosphatase activities are observed. The major fatty acids are C15:0 iso 50.75% and C15:0 anteiso 24.05%. The polar lipids present are diphosphatidylglycerol, phosphatidylglycerol, phosphatidylethanolamine, phospholipids 1 and 2, unidentified aminophospholipid and glycolipid. The peptidoglycan type is A4αL-Lys←D-Glu (type A11.33). Cells are susceptible to penicillin G, amoxicillin, amoxicillin plus clavulanic acid, imipenem, gentamycin, erythromycin, doxycycline, rifampicin, vancomycin and nitrofurantoin. The genome is 2,975,103 bp long with a 38.21% G+C content. A 36.3 kb prophage, KS1, was identified. The type strain is JC8E^T^ (= CSUR P138^T^ = DSM 24641^T^). The 16S rRNA gene sequence was deposited in GenBank with the accession number JF824796. The whole genome shotgun sequence of *K. senegalensis* strain JC8E^T^ was deposited in GenBank/DDBJ/EMBL under accession number CAEW01000000.
